# Advances of Spectrometric Techniques in Food Analysis and Food Authentication Implemented with Chemometrics

**DOI:** 10.3390/foods9111550

**Published:** 2020-10-27

**Authors:** Ioannis K. Karabagias

**Affiliations:** Laboratory of Food Chemistry, Department of Chemistry, University of Ioannina, 45110 Ioannina, Greece; ikaraba@uoi.gr; Tel.: +30-697-828-6866

**Keywords:** *Arbutus unedo*, coffee, honey, meat, *Pleurotus* mushrooms, oilseeds, rapeseed oil, chemometrics

## Abstract

Given the continuous consumer demand for products of high quality and specific origin, there is a great tendency for the application of multiple instrumental techniques for the complete characterization of foodstuffs or related natural products. Spectrometric techniques usually offer a full and rapid screenshot of products’ composition and properties by the determination of specific bio-molecules such as sugars, minerals, polyphenols, volatile compounds, amino acids, organic acids, etc. The present special issue aimed firstly to enhance the advances of the application of spectrometric techniques such as gas chromatography coupled to mass spectrometry (GC-MS), inductively coupled plasma optical emission spectrometry (ICP-OES), isotope ratio mass spectrometry (IRMS), nuclear magnetic resonance (NMR), Raman spectroscopy, or any other spectrometric technique, in the analysis of foodstuffs such as meat, milk, cheese, potatoes, vegetables, fruits/fruit juices, honey, olive oil, chocolate, and other natural products. An additional goal was to fill the gap between food composition/food properties/natural products properties and food/natural products authenticity, using supervised and non-supervised chemometrics. Of the 18 submitted articles, nine were eventually published, providing new information to the field.

## General Remarks

Monitoring food and natural products quality, on the basis of highlighting specific properties of foodstuffs and natural based products, and at the same time detecting and preventing adulteration and fraud, is an important topic today at international level. Therefore, there is a challenging need for the development of rapid and validated analytical techniques such as gas chromatography coupled to mass spectrometry (GC-MS), inductively coupled plasma optical emission spectrometry (ICP-OES), isotope ratio mass spectrometry (IRMS), nuclear magnetic resonance (NMR), Raman spectroscopy, or any other spectrometric technique, for screening the authenticity and fraud of foods and natural products in combination with chemometrics, which offer the analyst the ability for the deep characterization of such matrices. The present special issue aimed to cover such demands. Let us go deeper through the published articles in this special issue.

The application of High-Performance Thin-Layer Chromatography (HPTLC) analysis and Liquid Chromatography High Resolution Mass Spectrometry (LC–HRMS), coupled with Principal Component Analysis (PCA) by Maldini et al. [1], was effective for the discrimination of *Arbutus unedo* plant material (leaves, yellow fruit, and red fruit) collected from La Maddalena and Sassari (Sardinia, Italy).The use of HPTLC and PCA comprised a simple and reliable untargeted approach to rapidly discriminate extracts based on tissues and/or geographical origins, while that of LC–HRMS in combination with PCA could be used as an effective approach for the identification of specific metabolites (quercetin, kaempferol, and myricetin derivatives, chlorogenic acid, arbutin, etc.) that werecapable to discriminate samples.

A validated ^1^H NMR spectroscopic method was implemented for the routine screening of coffee quality and authenticity by Okaru et al. [2]. A factorial experimental design was used to investigate the influence of the NMR device, extraction time, and origin of coffee on the content of specific metabolites such as caffeine, 16-O-methylcafestol (OMC), kahweol, furfuryl alcohol, and 5-hydroxymethylfurfural (HMF) determined in coffee. The aforementioned method was successfully validated for specificity, selectivity, sensitivity, and linearity of detector response. The proposed method produced a satisfactory precision for all analytes in roasted coffee, except for kahweol in canephora (robusta) coffee. The proposed validated method may be used for routine screening of roasted coffee quality and authenticity control (i.e., arabica/robusta discrimination), as its applicability was demonstrated during the recent OPSON VIII Europol-Interpol operation on coffee fraud control.

Sikorska et al. [3] managed to test the usability of fluorescence spectroscopy to evaluate the stability of cold-pressed rapeseed oil during storage. The quality deterioration of oils was evaluated on the basis of several chemical parameters (peroxide value, acid value, K_232_ and K_270_, polar compounds, tocopherols, carotenoids, pheophytins, oxygen concentration) and fluorescence, concerning the freshly-pressed rapeseed oil that was stored in colorless and green glass bottles, exposed to light and in darkness, for a period of 6 months. Parallel factor analysis (PARAFAC) of oil excitation-emission matrices revealed the presence of four fluorophores that showed different evolution throughout the storage period. The fluorescence study provided direct information about tocopherol and pheophytin degradation and revealed the formation of a new fluorescent product. Principal component analysis (PCA) applied on the analytical and fluorescence data showed that oxidation was more advanced in samples exposed to light due to the photo-induced processes; only a very minor effect of the bottle color was observed. Multiple linear regression (MLR) and partial least squares regression (PLSR) on the PARAFAC scores revealed a quantitative relationship between fluorescence and some of the chemical parameters (tocopherols and pheophytins).

Karabagias et al. [4] presented a research study that comprised the second part of a new theory related to honey authentication based on the implementation of the honey code and the use of chemometrics. Chestnut, citrus, clover, eucalyptus, fir, pine, and thyme honeys from Egypt, Greece, Morocco, Portugal, and Spain were subjected to gas chromatography coupled to mass spectrometry (GC-MS) analysis in combination with headspace solid-phase microextraction (HS-SPME). The application of classification and dimension reduction statistical techniques (multivariate analysis of variance, linear discriminant analysis, *k*-nearest neighbors, and factor analysis) were applied to the semi-quantitative data of volatile compounds. Results showed that honey samples could be distinguished effectively according to both botanical origin and the honey code (*p* < 0.05), with the use of hexanoic acid ethyl ester, heptanoic acid ethyl ester, octanoic acid ethyl ester, nonanoic acid ethyl ester, decanoic acid ethyl ester, dodecanoic acid ethyl ester, tetradecanoic acid ethyl ester, hexadecanoic acid ethyl ester, octanal, nonanal, decanal, lilac aldehyde C (isomer III), lilac aldehyde D (isomer IV), benzeneacetaldehyde, *alpha*-isophorone, 4-ketoisophorone, 2-hydroxyisophorone, geranyl acetone, 6-methyl-5-hepten-2-one, 1-(2-furanyl)-ethanone, octanol, decanol, nonanoic acid, pentanoic acid, 5-methyl-2-phenyl-hexenal, benzeneacetonitrile, nonane, and 5-methyl-4-nonene. New amendments in honey authentication and data handling procedures based on hierarchical classification strategies (HCSs) were exhaustively documented in the aforementioned study, supporting and flourishing the state of the art.

In the study of Louppis et al. [5], asfaka, fir, flower, forest flowers, and orange blossom honeys harvested in the wider area of Hellas by professional beekeepers were subjected to mineral content analysis using inductively coupled plasma optical emission spectrometry (ICP-OES). In total, 25 minerals were identified (Ag, Al, As, B, Ba, Be, Ca, Cd, Co, Cr, Cu, Fe, Hg, Mg, Mn, Mo, Ni, Pb, Sb, Se, Si, Ti, Tl, V, and Zn) and quantified. The mineral content varied significantly (*p* < 0.05) according to honey botanical origin, whereas lead, cadmium, and chromium contents ranged between 0.05–0.33 mg kg^−1^, <0.05 mg kg^−1^, and in the range of <0.12 to 0.39 mg kg^−1^, respectively. Fir honeys from the Aitoloakarnania region showed the highest mineral content (182.13 ± 71.34 mg kg^−1^), while flower honeys from Samos Island recorded the highest silicon content (16.08 ± 2.94 mg kg^−1^). Implementation of multivariate analysis of variance, factor analysis, linear discriminant analysis, and stepwise discriminant analysis led to the perfect classification (100%) of these honeys according to botanical origin with the use of Al, As, Ca, Mg, Mn, Ni, Pb, Sb, Si, Zn and total mineral content. However, the higher lead content in the majority of samples than the recent regulated upper limit (0.10 mg kg^−1^), sets the need for further improvements of the beekeepers’ practices/strategies in honey production.

At the same time, honey adulteration comprises a major issue in food production, which may reduce the effective components in honey and, thus, has a detrimental effect on human health. The application of laser-induced breakdown spectroscopy (LIBS) combined with chemometrics was used by Peng et al. [6] for the rapid quantification of the adulterant content. Two common types of adulteration, including mixing acacia honey with high fructose corn syrup (HFCS) and rape honey, were quantified with univariate analysis and partial least squares regression (PLSR). In addition, the variable importance was tested with univariate analysis and feature selection methods (genetic algorithm (GA), variable importance in projection (VIP), selectivity ratio (SR)). The results indicated that emissions from Mg II 279.58, 280.30 nm, Mg I 285.25 nm, Ca II 393.37, 396.89 nm, Ca I 422.70 nm, Na I 589.03, 589.64 nm, and K I 766.57, 769.97 nm had compact relationship with the adulterant content. The most effective models for detecting the adulteration ratio of HFCS 55, HFCS 90, and rape honey were achieved by (SR-PLSR), (VIP-PLSR) and VIP-PLSR combined with root-mean-square error (RMSE) of 8.9%, 8.2%, and 4.8%, respectively. The study provided a fast and simple approach for detecting honey adulteration with cheap sweeteners.

In the study of Jiang et al. [7], a hyperspectral imaging (HSI) methodology was proposed to identify and visualize this kind of jowl meat adulteration in pork. Numerous hyperspectral images were acquired from adulterated meat samples in the range of 0–100% (*w/w*) at 10% increments using a visible and near-infrared (400–1000 nm) HSI system in reflectance mode. Mean spectra were extracted from the regions of interests (ROIs) and represented each sample accordingly. The performance comparison of established partial least square regression (PLSR) models showed that spectra pretreated by standard normal variate (SNV) performed best, with R_p_^2^ = 0.9549 and residual predictive deviation (RPD) = 4.54. Furthermore, functional wavelengths related to the identification of adulteration were individually selected using methods of principal component (PC) loadings, two-dimensional correlation spectroscopy (2D-COS), and regression coefficients (RC). The multispectral RC-PLSR model exhibited the most satisfactory results in prediction set that the R_p_^2^ was 0.9063, the RPD was 2.30, and the limit of detection (LOD) was 6.50%. Spatial distribution was visualized based on the preferred model, and adulteration levels were clearly discernible. The visualization was further verified that prediction results well matched the known distribution in samples. Overall, HSI was found to be a promising methodology for detecting and visualizing minced jowl meat in pork.

Furthermore, oilseeds from five native plant species with edible potential from the Brazilian Caatinga semi-arid region (*Diplopterys pubipetala*, *Barnebya harleyi*, *Croton adamantinus*, *Hippocratea volubilis*, and *Couroupita guianensis*) were subjected to mineral content analysis as reported in the study of M.C. Almeida et al. [8]. The minerals, Na, K, Ca, Mg, Fe, Cu, Cr, and Al, were analyzed by high-resolution continuum source atomic absorption spectrometry (HR–CS AAS) and P by the vanadomolybdophosphoric acid colorimetric method. The main elements found were K, Mg, and P (1.62–3.7 mg/g, 362–586 µg/g, and 224–499 µg/g on the basis of dry weight (dw), respectively). *B. Harleyi* seeds contained the highest amounts of K and P, while *C. guianensis* seeds were the richest in Mg. On the other hand, Fe was the most abundant oligoelement (2.3–25.6 µg/g dw). The Cr content was below the limit of quantification for all samples and Al content was low, ranging between 0.04–1.80 µg/g (dw). Linear discriminant analysis clearly differentiated *B. harleyi* and *C. guianensis* samples from the remaining ones. In sum, these oilseeds from the Brazilian Caatinga semi-arid region have the potential to be used as natural sources of minerals, mainly K.

Finally, attenuated total reflectance-Fourier transform infrared (ATR-FTIR) spectroscopy was used by Bekiaris et al. [9] to monitor the infrared absorption spectra of mushroom samples from *Pleurotus ostreatus*, *Pleurotus eryngii*, and *Pleurotus nebrodensis* strains, cultivated on wheat straw, grape marc and/or by-products of the olive industry. The spectroscopic analysis provided a chemical insight into the mushrooms examined, while qualitative and quantitative differences in regions related to proteins, phenolic compounds, and polysaccharides were monitoredamong the species and substrates studied. The use of advanced chemometrics, correlations of the recorded mushrooms’ spectra versus their content in glucans and ergosterol, commonly determined through traditional analytical techniques, allowed the development of models predicting such contents with a good predictive power (*R*^2^: 0.80–0.84) and accuracy (low root mean square error, low relative error, and representative to the predicted compounds spectral regions used for the calibrations). FTIR spectroscopy could then be exploited as a potential process analytical technology tool in the mushroom industry to characterize mushrooms and to assess their content in bioactive compounds.
